# “Out of our control”: Living through Cyclone Yasi

**DOI:** 10.3402/qhw.v9.19821

**Published:** 2014-01-15

**Authors:** Cindy Woods, Caryn West, Petra Buettner, Kim Usher

**Affiliations:** 1 School of Nursing, Midwifery & Nutrition, James Cook University, Cairns, Australia; 2 School of Public Health, Tropical Medicine and Rehabilitation Sciences, James Cook University, Townsville, Australia

**Keywords:** Australia, cyclone, mental health, disasters, emergency preparedness, qualitative research

## Abstract

The aim of this study was to explore the experiences of people who lived through Cyclone Yasi on 3 February 2011. Data from two open-ended questions (Q1: *n*=344; and Q2: *n*=339) within a survey completed by 433 residents of cyclone-affected areas between Cairns and Townsville, Australia, were analysed using a qualitative, thematic approach. Experiences were portrayed in three main themes: (1) *living in the mode of existential threat* describes survivors’ sense of panic and feeling at the mercy of nature as they feared for their life; (2) *unforgettable memories* describe feelings of emotional helplessness and the unimaginable chaos that the cyclone wrought; and (3) *centrality of others* shows how community support and closeness helped alleviate losses and uncertainty. A critical finding from this study was the negative role of the media in escalating fears for life prior to and during the cyclone, highlighting the need for government, community leaders, and health professionals to have a media plan in place to ensure that disaster warnings are taken seriously without inciting unnecessary panic. Although survivors experienced extreme vulnerability and a threat to life, the disaster also brought communities closer together and connected family, friends, and neighbours through the caring, support, and help they offered each other. This highlights the central role of others during the recovery process and underlines the importance of promoting and facilitating social support to aid recovery post disaster.

Disasters tend to strike suddenly and unexpectedly, but in the case of bush or wild fires, floods, and cyclones, a pre-impact warning period of hours or days may be available. This provides people with a window of opportunity to assess the risk, prepare their home and property, and decide whether to evacuate from the path of the disaster or to shelter in place. These decisions are often based on available information from trusted sources such as the government, the weather bureau, the media, friends, and family. People naturally seek information from multiple sources. They rely primarily on family and friends to validate and interpret information from formal sources, then collectively decide their response and possible evacuation (Mileti, Sorensen, Sorensen, & Sutton, 2002; Wachtendorf & Kendra, 2006).

Disasters are characterized by human and animal suffering and material damage (Roxberg, Sameby, Brodin, Fridlung, & Da Silva, 2010). When a disaster strikes, all members of a community are affected to varying degrees (Dass-Brailsford, 2008). In the immediate aftermath of a disaster—the acute post-disaster phase—stress, grief, depression, anxiety, and dissociative symptoms are all normal reactions to an abnormal situation (Cohen, 2002; Reyes & Elhai, 2004). The risk of an acute stress reaction increases when one's own life is threatened (Roxberg et al., 2010). Mental health symptoms are exacerbated and prolonged by stress, loss, relocation, lack of social support, and disruption to daily activities (Mitchell, Witman, & Taffaro, 2008; Nikapota, 2006; Norris et al., 2002). In the months following a traumatic disaster, acute reactions are replaced by more chronic psychological conditions; thus, ongoing mental health treatment is essential to community resilience and recovery (Madrid & Grant, 2008; Rosser, 2008; Vijaykumar, Thara, John, & Chellappa, 2006). As communities affected by disaster begin to recover and regain their normal pre-disaster conditions, psychological reactions also begin to return to normal functioning (Norris, 2005; Reyes & Elhai, 2004). However, in a small percentage of people, long-term psychological impacts of disasters can be experienced. These include post-traumatic stress disorder, substance abuse, and major depression (Leon, 2004; Usher, Brown, et al., 2012). Given this, there is a need to gain a holistic understanding of survivors’ experiences in natural disasters, and few studies have focussed on the lived experience of cyclone survivors. By studying this group, knowledge can be obtained that helps healthcare workers understand how lives are affected, especially in the medium to long term after a disaster period, to improve care and treatment. Furthermore, communities can use the findings to increase preparedness and strengthen resilience prior to disasters occurring.

Peoples’ lived experiences of a severe weather disaster such as a cyclone have rarely been described from an individual perspective. Previous research in the context of severe weather disasters has focussed mainly on analysing the individual experience from a psychological or psychiatric viewpoint, in which the occurrence of post-traumatic stress disorder or other mental health issues has been a central issue (Acierno et al., 2007; Norris, Perilla, Riad, Kaniasty, & Lavizzo, 1999; Norris & Uhl, 1993; Sattler et al., 2002; Wu, Yin, Xu, & Zhao, 2011). In contrast, a holistic understanding of cyclone survivors’ experiences is proposed as a way to better understand how cyclones affect peoples’ lives. These understandings can be used to develop strategies that can be used to lessen suffering, encourage resilience, and help survivors to move forward with their lives.

## Methods

### The study context

In the last days of January 2011, a cyclone warning was upgraded to a cyclone watch. A cyclone-tracking map predicted that the cyclone landfall site would be Cairns. Tropical Cyclone (TC) Yasi struck North Queensland, Australia, on Thursday, 3 February 2011, between midnight and 1 a.m. Australian Eastern Standard Time (Bureau of Meteorology (BOM), 2011). The damaging winds and rain wreaked havoc across the region, affecting an approximately 350 km stretch of coast between Townsville and Cairns. TC Yasi ranks as one of the most powerful cyclones to have crossed the coast of Queensland since 1918 (BOM, 2011). Fortunately, TC Yasi did not cause a direct loss of life; one death was indirectly attributed to TC Yasi from carbon monoxide poisoning due to operating a generator inside the home (ABC News, 2011). The impact of the cyclone on North Queensland, however, was profound in terms of infrastructure damage, damage to vegetation (including commercial crops), economic effects, and psychological distress.

### Data collection

Six to 12 months after TC Yasi devastated rural towns in North Queensland, a survey was made available to affected residents. The aim of this survey was to examine the steps taken by residents of the affected regions to prepare for this impending natural disaster and to identify their resource losses and symptoms of psychological distress following TC Yasi. The survey was available electronically through Survey Monkey and in hard copy at local libraries for those people unable to access the electronic version. Requests for residents to complete the survey were distributed through the local media, including newspapers and radio. Signs were posted at local community venues, like libraries and local markets, as well as through a web page hosted by the local university. A total of 433 responses were received, 223 (51.5%) electronically and 210 (48.5%) in hard copy. Hard-copy surveys were manually coded in Survey Monkey.

### Instrument

The survey tool was adapted from the one used by Sattler et al. (2002) to survey Hurricane Georges survivors. The survey consisted of 11 sections: the first contained demographic questions (eight questions); the second section asked questions about the timing of preparedness efforts, including one question that assessed the perception of level of preparedness (nine questions); section 3 (two questions) asked about property damage and insurance; section 4 (five questions) asked about loss of services and return to work or study; the fifth section (six questions) asked about personal characteristic resources (e.g., a sense of optimism and feeling that you have control over your life); section 6 (five questions) asked about condition resources, such as family stability and closeness with friends; section 7 (three questions) addressed object resource loss (sentimental possessions and transportation); section 8 (two questions) questioned energy resources, free time, and time for sleep; section 9 (three questions) asked about the object resources food, medication, and money; section 10 comprised five questions related to acute stress disorder and/or post-traumatic stress disorder (e.g., *I avoid things that remind me of the cyclone*); and the final section had two open-ended questions about overall thoughts about cyclone preparation and current feelings about the cyclone.

A total of 344 (79.4%) participants responded to the first open-ended question, *What are your overall thoughts about your preparation for Cyclone Yasi?* and 339 (78.3%) participants responded to the second open-ended question, *What are your feelings now about Cyclone Yasi?* Responses ranged from one or two sentences to short paragraphs. This article focusses on the themes that emerged from these two open-ended questions. The results from the remainder of the survey are reported elsewhere (Usher, Buettner, et al., 2013).

### Data analysis

Thematic analysis has the advantage of theoretical freedom and offers the potential to provide a rich and detailed account of the data (Braun & Clarke, 2006). There was no pre-existing coding framework or assumed sub-topics for the investigation; such assumptions may have overlooked important parts of the participants’ experiences. Instead, an inductive approach was considered appropriate for the present study as this allowed the data to drive the analysis and allowed the emerging themes to be strongly linked to the data. The data were analysed using methods of thematic analysis.

Qualitative data analysis software Nvivo v10 was used to manage the large volume of data and assist with the organization of themes. Data from the two open-ended questions were extracted from Survey Monkey and imported into Nvivo. Data from all questionnaires were analysed. Two authors (CWo and CWe) read the survey responses several times to acquire a broad overview of the cyclone survivors’ experiences prior to, during, and after the cyclone. Repeated readings of the text led to the organization of meaning units that were condensed while preserving the fundamental meaning. The condensed text was then abstracted and described with a preliminary code. This was done by the first author (CWo), and any uncertainties or ambiguities were discussed with another author (CWe). Together, the two authors ensured that the coding represented the meaning units and the text as a whole. The codes were sorted into subthemes. Next, three main themes were created based on the content of subthemes, the text as a whole, and the interpretation of the underlying meanings (Table I) (Graneheim & Lundman, 2004).

**Table I:** Examples of the analysis.

Meaning unit	Condense	Code	Sub-theme	Theme
I watched the news for a bit and had to turn it off cause I was terrified of what they were saying even though I was very organised, using words like killer, deadly etc. They really had a lot to answer for.	Not happy with the media.	Media hype	Social amplification of risk	
It was the most terrifying experience of my life. We took refuge in a little, unfinished besser block shelter on the property (no shower or toilet) that we thought would be reasonably well protected as there is a natural ridge behind it. Our little Jack Russell pup was the first to show signs of being frightened, and his anxiety, along with mine just grew and grew, like the noise which was the hardest thing for me to deal with. Unlike Larry, where we could see what was happening for the most part—this helped me to cope funnily enough. However, Yasi's full force came later, during the night when it was pitch black and you couldn't see anything. That was the hardest thing for me to deal with, and the noise; like a freight train boring down on you.	Anxiety and terror	Fear for life	At the mercy of nature	Living in the mode of existential threat
I cannot talk about the damage to the forest and our garden without crying that sums it up.	Damage to environment	Emotional aftermath	Stripped naked	

Identified themes were investigated in relation to recent literature concerning disasters and mental health. These themes were considered in terms of their broader meanings and implications for emergency services personnel, disaster management groups, and mental health workers post disaster. Internal logic and reliability are established by quotations from the text (Polit & Beck, 2006). The identifiers in front of each quotation in this article denote the participant's sex (M or F), age group, and community of residence. Only one participant was quoted twice; all other quotations are from a variety of survey respondents. Each subtheme discussed in this article is representative of a majority of responses for that particular subtheme. The findings are assumed to be transferrable to similar contexts where people experience a severe weather disaster, but they are particularly relevant to survivors of a cyclone, hurricane, or typhoon.

## Ethical considerations

This study was conducted in accordance with the principles outlined in the Declaration of Helsinki and the National Health and Medical Research Council (NHMRC), and it was approved by the Human Ethics Research Committee at James Cook University (HREC H4116). Information about the research project and a request to participate were given to cyclone-affected residents. They were informed that participation was voluntary and that they had a right to withdraw at any time without explanation. Residents who agreed to participate gave informed consent before completing the survey. For those who completed the online questionnaire, information about the survey was on the first page of the online questionnaire, and consent was implied by completion of the questionnaire.

## Results

The 433 survey respondents were residents of towns that were affected by TC Yasi and located between Cairns (*n*=139; 32.1%) and Townsville (*n*=62; 14.3%), in particular the coastal communities that bore the main impact of TC Yasi: Babinda (*n*=12; 2.8%), Innisfail (*n*=42; 9.7%), Mission Beach (*n*=72; 16.6%), Tully (*n*=52; 12.0%), Cardwell (*n*=35; 8.1%), and Ingham (*n*=19, 4.4%). Of the 433 respondents, 323 (74.6%) were female. The age distribution of respondents was bimodal, with 17.8% aged 18 to 24 years and 36.3% aged 45 to 59 years. The majority of respondents identified as Caucasian (90.8%), and eight respondents (1.8%) were of Australian Aboriginal and/or Torres Strait Islander descent.

Analysis of the text revealed three themes that illuminate the survivors’ experiences from a cyclone: (1) living in the mode of existential threat, (2) unforgettable memories, and (3) centrality of others. Themes and subthemes are illustrated with quotations.

### Living in the mode of existential threat

This theme explores survivors’ experiences prior to the cyclone, during the cyclone, and in the immediate aftermath of the cyclone. The participants described how, in the days immediately prior to the disaster, they were thrown into a sense of panic and felt they may not survive the approaching threat. As the cyclone crossed the coast, they felt they were at the mercy of nature and wondered if they would survive. Post-cyclone survivors felt stripped naked, leaving only raw emotions in the chaotic aftermath.

#### Social amplification of risk

As North Queensland residents absorbed the implications of the advancing cyclone, media reports amplified the extent of the threat with headlines such as “in killer's eye,” “worst storm in history,” “monster at the door,” and “mother of all storms.” A sense of the ordinary evaporated as supermarket shelves were stripped bare in a spate of panic buying and as long queues formed for commodities such as ice, gas, and petrol:

(F 40–44 Mission Beach) I think, whilst warnings are important, that TV went overboard and the dire warnings increased stress levels and in some cases led to panic. I feel I could have been better prepared.

Fear of the impending cyclone was manifest in the thousands of people queuing at the airport trying to leave the area. The stress and anxiety generated by media reports and the actions of others were infectious, and those unable or unwilling to leave the area feared for their lives.

(F 35–39 Innisfail) I think due to the sheer size of the cyclone and the hype from the media, the experience leading up to this cyclone was very scary. I remember reading the BOM site and the words about worst in generations and catastrophic was gut-wrenching. I remember I could not stop crying and shaking.

Social amplification of risk by media outlets enhanced the feeling of being trapped in a hopeless, life-threatening situation. The sense of impending catastrophe was increased by contact from terrified friends and relatives who thought their loved ones were going to die.

(F 35–39 Cairns) I was not happy with how the national media handled themselves. I had friends and family calling, crying, thinking we were going to die and very concerned for our well being. I watched the news for a bit and had to turn it off cause I was terrified of what they were saying even though I was very organised, using words like killer, deadly etc. They really had a lot to answer for.

#### At the mercy of nature

The survivors gave vivid descriptions of their experience of facing death before and during the cyclone. Evacuation centres filled to capacity quickly and had to turn people away (Brisbane Times, 2011; The Guardian, 2011). Those who chose to stay and shelter at home began to despair that they had made the wrong decision. Once survivors were in the midst of the cyclone, it was too late to leave or to shelter elsewhere; there were no emergency exits and no possibility of escape.

(F 65–69 Cairns) We were not told about the seriousness and the danger of death until it was too late to leave by public transport. We would have chosen to fly south rather than die in our home unable to leave. I did not want to die in the tidal surge and not be found for my family to bury. We should have been given a choice. We said our goodbyes and prepared for death.

In a relatively short time, the well-known world was blown away and survivors felt themselves to be at the mercy of nature. Survivors experienced enormous fear while enduring the “endless” night.

(F 60–64 Tully) The feeling of being at the mercy of nature at her worst was the most and, still is, terrifying. I am shaken to the core of the instability and fragility of life in a situation out of our control.

During the long night of the cyclone, there was no power, and survivors could not see the violent forces of nature outside. This darkness which hid the cyclone from view and the relentless noise of cyclonic winds escalated deep anxiety and the experience of proximity to death.

(F 50–54 Tully) It was the most terrifying experience of my life. We took refuge in a little unfinished besser block shelter on the property (no shower or toilet) that we thought would be reasonably well protected as there is a natural ridge behind it. Our little Jack Russell pup was the first to show signs of being frightened, and his anxiety, along with mine, just grew and grew like the noise, which was the hardest thing for me to deal with. Unlike Larry, where we could see what was happening for the most part—this helped me to cope funnily enough. However, Yasi's full force came later, during the night when it was pitch black and you couldn't see anything. That was the hardest thing for me to deal with, and the noise, like a freight train boring down on you.

Fear and uncertainty overwhelmed survivors, and the threat to their safety was described as a form of living hell:

(F 30-34 Mission Beach) Was bigger and more frightening having based our mental readiness on Larry which was a walk in the park. This was the sport of Satan to say the least.

#### Stripped naked

The morning after the cyclone, survivors were faced with unimaginable chaos when they looked outside. The world had turned into an unrecognizable landscape that did not match their memory. Many trees had fallen, while others had lost branches and were stripped of foliage. Fences had been knocked down, outbuildings were damaged, and, for some people, their houses were destroyed. Shock at the extent of the damage and the alien landscape left survivors feeling stripped naked with only raw emotions in the chaotic aftermath.

(F 70–74 Tully) We live at the foot of Mt … and overlook the valley. The morning after Yasi passed and I looked out over the valley, not only had the valley been stripped naked, I felt naked also. It's the only way I can describe how I felt. The helplessness, the joy of everyone being safe, the utter destruction we were looking at and the wondering of how on earth we were to go about doing what had to be done. My sister and husband's house at … was completely destroyed that was heart wrenching and a deep pain felt by us all.

Overnight, the survivors’ world was completely changed, leaving an unknown world in its place that would never be the same. For the survivors, it was a struggle to come to terms with this new world: (F 50–59 Innisfail) “I will feel worse if we have another one soon, this one is still raw and still being coped with.”

### Unforgettable memories

This theme explores the days, weeks, and months following the cyclone. Memories of surviving a disaster affected participants’ lives in differing ways. The participants described their emotions as they tried to pick up the pieces of their lives and cope with the chaos the cyclone had wrought. For some, living through the cyclone was a testing and life-affirming experience, while, for others, their cries for help were unheard.

#### Emotional helplessness

In the aftermath of the cyclone, survivors struggled to understand and to come to terms with their experience and their losses. The feeling of not being in control of their lives or their destiny, and of being at the mercy of a fearsome and violent force of nature, left survivors with a sense of emotional helplessness:

(F 50–54 Mission Beach) That it is a natural event. And that these types of events can bring about depression if you felt that you had no control of your life previous to the cyclone. I was surprised to how childhood circumstances that had been buried were brought to the surface, and the emotional helplessness resulting from the feeling you have no control over your life in general. After several months I sought help.

For some, the period after the cyclone was like being in a nightmare that one could not wake from:

(F 35–39 Mission Beach) It makes me very sad to see the devastation to the rainforest, despite the knowledge that it is growing back because it took so long to grow back from Cyclone Larry, and was still recovering, when Yasi hit. I'm sad to see such a beautiful town as Mission Beach with half-mast trees and damaged shorelines. I love living at Mission—and have done so for 11 years now but I'm ever fearful of another big cyclone now. I don't feel like you can dismiss two big cyclones in a row as flukey or unlucky.… I like to think of myself as a positive person but Cyclone Yasi is a bad memory and one that hasn't really been relegated to “memory” yet because we're still living it.

Memories of the intense fear that was felt on the night of the cyclone continued to haunt survivors for many months after the event:

(F 50–54 Ingham) I will never forget how terrified we were but thankful that our home was not destroyed even though we lost our fences, garden shed and trees and hope that nothing like Yasi will ever hit Ingham again.

Survivors described experiencing acute stress symptoms and depression, and feeling disorientated, lost, and emotionally overwhelmed:

(F 50–54 Cardwell) I feel teary from time to time. Am on a waiting list to see a psyche [psychiatrist/psychologist] and am on anti-depressants.

#### Going beyond limits

While some people felt haunted by the past, others spoke of how surviving the cyclone was a life-affirming experience. Survivors were reminded of the fragility of life and consequently appreciated life more than before the cyclone. To battle the elements and come through the traumatic event were described as finding an inner strength and resilience that were unknown before:

(F 55–59 Innisfail) It was a time of growing and maturing and to go beyond limits. Personally, I was not greatly affected as far as losing belongings is concerned. Since my belongings are not “my life.” … I would still be ok if I had lost everything. Being reminded what life is all about and our vulnerability and mortality as an individual is a good thing (I think:-)). I'll be back to help with the next one!

#### Unheard cries for help

In the aftermath of the cyclone, townships were isolated by flooding, and fallen trees blocked roads. Survivors living outside of the main townships spoke of feeling isolated and neglected as they endured a wait of up to a week for vital supplies and assistance.

(M 50–54 Cardwell) Quite a lot of resources arrived quite quickly for the main town areas, which was necessary because most people are concentrated in those areas, and they did need help. BUT so did the other people not living in the immediate town area, many of those areas were largely forgotten for quite some time and had to fend for themselves for more than a week after the cyclone. Resources were not being distributed to outlying areas even though they suffered loss and damage to as great a level as those in the towns.

### Centrality of others

At the eleventh hour, the cyclone veered south instead of crossing land over a major regional city. The cyclone hit a number of small coastal towns. This theme explores participants’ feelings of relief at being spared the worst of the storm, but at the same time, feeling great sorrow and sympathy for those who were hit hardest—those whose homes were destroyed and who lost everything. Community support and closeness gave participants a sense of wellbeing and helped alleviate their losses and uncertainty, underlining the importance of other people in the process of coming to terms with their experience.

#### Getting a second chance

After the cyclone, media reports showed graphic images of the devastation and destruction that occurred in the hardest-hit areas. Media reported that the major regional cities “dodged a bullet” and fortunately escaped the major force and impact of the cyclone. After being overwhelmed by uncertainty as to whether they would survive the cyclone or not, participants who resided on the periphery of the main impact zone expressed how lucky they had been to avoid a direct hit.

(F 25–29 Cairns) I feel that my family and I were very lucky, receiving only minor damage to our farm infrastructure - mostly from falling trees onto fences. I feel we were very fortunate to not have received the full brunt of the cyclone. If we had, it would have been devastating. I truly feel for those that were at the centre of the cyclone and value the experience that we were spared.

If landfall had coincided with high tide, a tidal surge of at least 5 m would have occurred, resulting in extensive flooding of the two regional cities on the periphery of the impact zone. Many residents were evacuated from low-lying coastal areas, and some flooding did occur, but participants were cognizant that the impact could have been much greater:

(F 70–74 Cardwell) Don't want to see another one like it. It was the biggest ever to hit this country. Could have been a lot worse if it had hit at high tide. We were lucky.

After suffering deep anxiety and fear of dying, the feeling of having cheated death and surviving was a huge relief: (F 25–29 Townsville) “It was a force of nature and we were extremely lucky not to have a huge fatality rate.”

#### Sadness and sympathy

Participants expressed profound sorrow and sympathy for people who were badly affected by the cyclone. They had lived through the same life-threatening cyclone, albeit on the fringes, and had their own experiences of chaos but recognized that others fared far worse than they did: (F 18–24 Cairns) “I feel extreme sympathy for those who were affected in a negative way.” By focussing on other people, participants were able to draw the focus away from themselves and feel thankful that they were relatively unscathed by the cyclone.

(F 55–59 Ingham) Don't want to go through it again. Life more precious, material things don't matter, don't buy things for fun anymore, don't want them. Get upset thinking of going through it again, frightened of loss of loved ones, feel sad for people with destroyed homes.

Participants also recognized and empathized with the psychosocial effects that the cyclone had wrought in affected communities. (F 70–74 Tully) “I feel a sense of profound sadness for many people who are suffering depression.”

#### Sense of community

In the aftermath of the disaster, the hardest-hit communities rallied together and provided support for one another: (F 18–24 Cardwell) “The community came together and helped one another. It has made the town's people closer.” The need to talk to others who had the same experiences and to provide and receive social support was important for survivors:

(F 65–69 Townsville) While I had no phone I visited friends (since I did have a car and petrol) to check on their welfare. I also caught up with people I'd not seen in a while when we all met up at our daily trip to the ice works.

The cyclone had some positive effects in that participants felt people were more open to friendship and support; they had the opportunity to spend time with relatives and overcame the cocooning effect that tends to occur in cities where people are isolated and generally do not know their neighbours:

(F 50–54 Townsville) However, there were positive outcomes in that the Cyclone brought all our immediate family members back together under one roof for a few days, along with their partners, and this was a unique situation that we all enjoyed. We also communicated and worked with neighbours, when normally everybody is “cocooned.” This working/sharing together bonding phenomenon was also evident at a local community level, and even to some extent at a state and national level.

The experience also brought out the best in people. Selfless acts were commonplace as everybody wanted to help those in need: (M 70–74 Cardwell) “I have been delivering food parcels to those in need.” Volunteerism became the norm during this time of crisis: (M 18–24 Cairns) “As a student, I had the opportunity to help at the hospital.” The cyclone brought the communities closer and connected family and friends through the caring, support, and help they offered each other.

## Discussion

The aim of this study was to explore cyclone survivors’ personal lived experiences and feelings. The findings revealed that the following themes—living in the mode of existential threat, unforgettable memories, and the centrality of others—shed new light on the survivors’ experiences. Living in the mode of existential threat involved social amplification of risk, feeling at the mercy of nature, and stripped naked of all but raw emotions. Survivors were critical of the media for spreading panic and fear locally, nationally and internationally.

Media reports can amplify and distort disaster information by focussing on the sensationalist aspects of an impending threat, and emphasizing the potential risk of harm and damage rather than precautions, preparedness, and mitigation activities (Kasperson et al., 1988; Tierney, Bevc, & Kuligowski, 2006). Distorted media reports create a ripple effect as individuals and groups react strongly, causing further amplification of the perceptions of risk and impact and generating unnecessary fear and panic (Barnes et al., 2008).

So great was the media focus on the threat to life that friends and relatives nationally and internationally were contacting loved ones in North Queensland, afraid they were going to die. Hearing this fear from others created a ripple effect and amplified the fear experienced by those individuals threatened by the approach of Cyclone Yasi (Barnes et al., 2008).

Feeling at the mercy of nature resonates with previous research about survivors of a train crash and of the 2004 Asian tsunami, which showed that the greatest impact on survivors was the experience of facing death and feelings of loss of control (Forsberg & Saveman, 2011; Råholm, Arman, & Rensfeldt, 2008). For those who were unable to evacuate, or who chose to shelter in place, fear of life dominated feelings while total chaos raged outside. After the cyclone passed, shock, sadness, and raw emotion were felt because of the destruction and changed appearance of the environment. This finding indicates that survivors experienced *solastalgia*, a form of homesickness similar to nostalgia, where the environment changes so much that it has a negative effect on people's quality of life and engenders a longing for their home environment to be the way it was (Albrecht, 2005; Albrecht et al., 2007).

The theme of *unforgettable memories* followed two paths. Some survivors experienced emotional helplessness, had difficulties overcoming their traumatic memories, and suffered stress and depression. Other survivors perceived the disaster to be a life-affirming experience and discovered a previously unknown inner strength and resilience. The negatively affected survivors were afraid of another cyclone and felt anxious as the next cyclone season approached. Individuals are affected by a traumatic event in varying degrees over time (Bonanno, Galea, Bucciarelli, & Vlahov, 2006). Experiencing a threat to life is a predictor of post-traumatic stress disorder, as is the extent of the disaster, the amount of devastation, and the disaster's overall impact on usual daily activities, which may explain why some survivors have difficulty overcoming their memories and moving forward (Leon, 2004; Ozer, Best, Lipsey, & Weiss, 2003). The positive view taken by other survivors is supported by studies that reported personal growth following trauma and adversity (Linley & Joseph, 2004; McMillen, Smith, & Fisher, 1997; Tedeschi & Calhoun, 1995; Updegraff & Taylor, 2000).

Survivors living outside of the main cyclone-affected townships expressed a sense of isolation and neglect. In the immediate aftermath of a cyclone, it may be impossible for people who need help to leave their homes due to fallen trees, flooding, damage to vehicles, injuries, or restricted mobility. Additionally, support services for elderly and disabled people (e.g., personal carers, blue nurses, and meals-on-wheels groups) may not be able to provide support if they too have experienced losses and emotional impacts as a result of a disaster. During this period, when people are in most need of help, roads are often blocked and/or flooded, telephone communication fails, and support people may be unable to reach those people for whom they care. Although it is logical to focus help and resources in areas with the highest population, the result may be that people in urgent need of physical or emotional support and resources are overlooked and do not receive sufficient help in the immediate aftermath of a disaster. We suggest that although the main recovery effort is located in the townships, a mobile recovery team, including locals who know the area, should conduct a timely outreach to all outlying farms and homes in disaster-affected areas to assess needs and offer support and resources. This could be prepared well in advance when dealing with approaching disasters. In rural towns, a system could be set up that registers the number of people, children, and people living with disability or medical conditions. The register could include vulnerable groups’ and individuals’ distance from the township. Installing and maintaining communication with these people from a central place in town during and after the disaster would ensure that needs can be heard and met.

While many survivors considered themselves fortunate that the cyclone changed direction and they escaped the worst of the storm, survivors also expressed sadness and sympathy for those who were in the path of the cyclone. After the cyclone, graphic images of destroyed homes, traumatized homeowners, and environmental damage were shown on television and in newspapers. Prior research has shown that individuals indirectly exposed to collective stress, primarily through media images and text, have increased vulnerability to post-traumatic stress symptoms (Clarke, 2008; Holman, Lucas-Thompson, & Lu, 2011; Osofsky, 2008). However, the opportunity to talk and share fears and feelings with a support network can be beneficial and can lower the risk of post-traumatic stress symptoms (Holman et al., 2011). Survivors perceived strong community support both before and after the disaster, which had a positive effect on their recovery, a finding supported by prior research which demonstrated that people with strong family or social support networks fared better post disaster than those without strong family or friendship networks post disaster (Acierno et al., 2007; Janoff-Bulman, 1992; Norris, Smith, & Kaniasty, 1999; Sattler et al., 2002). The findings from this study indicate that interaction with others and interdependence play important roles in recovery. Therefore, closeness and connections with social support networks should be encouraged and facilitated by cyclone recovery teams (Forsberg & Saveman, 2011).

## Limitations

The results of this study may have been different if more men had participated in the study, given that the survey respondents were predominately women (74.6%). Women are at higher risk of reporting post-traumatic stress symptoms after they experience a traumatic event (Berg Johannesson, Michel, Arnberg, & Lundin, 2006). However, women are more likely to experience greater levels of positive personal growth post disaster (Linley & Joseph, 2004; McMillen, Smith, & Fisher, 1997; Tedeschi & Calhoun, 1995; Updegraff & Taylor, 2000). Possibly, some people avoided participation because they were still feeling deeply affected by the disaster. Conversely, some people may have avoided participation because they were on the periphery of the cyclone and less affected and did not feel they had much to contribute to the study. According to Hussain, Weisaeth, and Heir (2009), non-participation is usually related to lack of interest or the feeling that one has nothing of interest to contribute. Therefore, it is possible that this account of cyclone experiences is limited because those people chose not to participate.

The survey was conducted 6 to 12 months after the cyclone and relied on retrospective recall of events, feelings, and experiences. While the chaos and distress in affected communities immediately after the disaster prevented earlier data collection, responses may have been different if the survey had been conducted in the weeks or months immediately following the cyclone. While these are significant limitations, the experiences, thoughts, and concerns expressed by the survivors in this study provide insight into their lived experience of a cyclone. Knowledge of peoples’ experiences and post-disaster impacts may help emergency and disaster planners, and health professionals who interact with cyclone survivors, to better address their concerns.

## Conclusions

The pre-impact warning stage of a disaster is an important time when information needs to be conveyed in such a way that it motivates people to take the impending threat seriously. People need to prepare and take precautions, but it is not in the best interest of communities to be instilled with overwhelming fear and panic. Headlines such as “killer cyclone” and “mother of all storms” and descriptions such as “catastrophic monster” create a sense of hopelessness and helplessness. Feelings of loss of control over life may be exacerbated in an already concerned population (Råholm et al., 2008). It is imperative that, in the event of a disaster, the government, the weather bureau, and public health professionals have a media plan that communicates risk without using hyperbole. The strategy needs to communicate preparedness and precaution strategies, but also to avoid widespread panic and fear (Barnes et al., 2008). Much of the fear and panic that occurred in the lead-up to the cyclone could have been avoided if media messages did not overtly focus on the worst-case scenario, death, and destruction.

The current study has shown that loss, crisis, and trauma are experienced by people in afflicted communities in all stages of a cyclone disaster—the pre-impact warning stage, during a cyclone, and in the acute stage immediately post disaster. People in areas of lower intensity impact also experience these negative feelings. It is important that post-disaster mental health programs and outreach services recognize and support the needs of people in the periphery of the main impact zone. In the immediate post-disaster stage, when recovery teams enter disaster-affected areas, it is recommended that mobile recovery teams are deployed to ensure that those survivors in outlying areas receive vital support and resources. Social support networks of family, friends, neighbours, and community members all have a positive effect on post-disaster recovery and wellbeing, and contact with social support networks should be encouraged and facilitated after a disaster, particularly for people who live outside of urban areas. Natural disasters are often frightening events and difficult for us to understand because they strike indiscriminately and we have no control over them. They can bring sorrow and devastation, but they can also become life-changing experiences that teach important lessons about what is important and about the fragility and joy of life.
